# The Effect of Temperature on the Phenotypic Plasticity of the Invasive Perennial Weed *Ambrosia confertiflora*

**DOI:** 10.3390/plants15020214

**Published:** 2026-01-09

**Authors:** Yifat Yair, Moshe Sibony, Yaakov Goldwasser, Hanan Eizenberg, Baruch Rubin

**Affiliations:** 1 RH Smith Institute of Plant Sciences & Genetics in Agriculture, Faculty of Agriculture, Food and Environment, The Hebrew University of Jerusalem, P.O. Box 12, Rehovot 76100, Israel; yairy2006@gmail.com (Y.Y.); moshe.sibony@mail.huji.ac.il (M.S.); rubin@mail.huji.ac.il (B.R.); 2 Department of Plant Pathology and Weed Research, Newe Ya’ar Research Center, Agricultural Research Organization, P.O. Box 1021, Ramat Yishay 30095, Israel; eizenber@volcani.agri.gov.il

**Keywords:** invasive weed, ragweed, thermal modelling, biomass allocation, climate change

## Abstract

The invasive perennial weed *Ambrosia confertiflora* (Burr ragweed) is widespread across various climatic regions in Israel and neighboring countries. This study examines how temperature affects the development of the plants’ aboveground and underground organs, as well as biomass allocation. We hypothesize that temperature influences how the plant distributes resources, thereby modifying its phenotypic morphology and contributing to its spread. Plants were grown in a phytotron under four seasonal temperature regimes (10–16 °C, 16–22 °C, 22–28 °C, 28–34 °C, N-D, 14 h light). We measured above- and belowground biomass, growth form, leaf size, and the interaction between temperature and apical dominance. Our results show that biomass allocation varies with temperature and developmental stage. During early growth, resources are primarily directed toward shoot development and leaf production. As plants matured, they shifted more resources to underground structures, eventually balancing allocation. At lower temperatures, plants invested more in underground growth while the shoot remained in the rosette form. In contrast, higher temperatures favored aboveground growth. *Ambrosia confertiflora* demonstrates significant phenotypic plasticity in response to temperature variation, affecting plant height, leaf morphology, and resource allocation in both shoot and underground tissues. Understanding how temperature drives these changes is critical to understanding the spread and ecological impact of this highly adaptable weed.

## 1. Introduction

The genus *Ambrosia*, part of the Asteraceae (Compositae) family, includes over 40 species [1,2]. Most species are native to North and South America, and these species spread to Europe, Asia, and Australia primarily through global trade.

In Israel, the native species *A. maritima* L. (sea ragweed) [3,4] likely became extinct, with the last recorded sighting in 1981 [5]. Other *Ambrosia* species arrived in Israel at various times [6], as documented in the National Herbarium of the Hebrew University of Jerusalem. The first arrivals were annual species: *A. artemisiifolia* (recorded in 1925, 1987, 1999, and 2013) and *A. trifida* (1964, 1987, and 2007). Perennial species appeared later: *A. tenuifolia* (1984, 1991), *A. confertiflora* (1990), *A. psilostachya* (1996, 2007), and *A. grayi* (2017). All the perennial species became naturalized, except *A. confertiflora*, which has become an invasive species in Israel [7,8].

*Ambrosia confertiflora* seeds require light to germinate and typically sprout from the soil surface, without any pre-emergence treatment. They can germinate year-round, though the majority of the seedlings (~40%) emerge during the fall, winter, and spring. In contrast, summer germination drops sharply to around 10% [8,9]. The weed is an erect plant, reaching up to 3 m in height. It produces a large number of achenes and rhizomes, causing rapid plant spread and establishment. It thrives in various habitats, such as riverbanks, roadsides, field crops, orchards, and disturbed areas [8,10].

Outside its native range in the southwestern United States and northern Mexico, *A. confertiflora* has so far spread only to Israel, the Palestinian Authority, Tunisia, and Australia [11,12]. The most widespread and well-studied species in this genus is *A. artemisiifolia*, which is invasive in Europe, North America, Asia, and Australia. In Israel, it has not spread widely due to the dry summers, but can survive in humid habitats, such as the Agamon nature reserve in the Hula valley [9].

Research on the response of *A. confertiflora* to environmental conditions is limited. In contrast, studies show that *A. artemisiifolia* displays high phenotypic plasticity, allowing it to adapt and thrive in diverse habitats and respond to climate change [13,14,15]. This plasticity can extend its pollen release season, increasing exposure to allergens, and potentially expanding the incidence of allergic rhinitis and asthma [16].

Global climate change, especially rising temperatures, may significantly alter ecosystems. These changes can promote the rapid spread and establishment of invasive species, causing ecological and economic damage beyond their native ranges [17].

Traits such as plant height, biomass production, and reproductive success (e.g., flowering time) vary in response to environmental factors. The phenotypic plasticity of *A. artemisiifolia* is shaped by environmental flexibility, genetic diversity, and interactions with different agricultural ecosystems [18]. While phenotypic plasticity supports adaptation, it can also involve trade-offs, such as reduced reproduction under extreme conditions, highlighting the delicate balance that invasive species must maintain in fluctuating environments. *Ambrosia artemisiifolia’s* ability to adapt to simultaneous changes in temperature and nutrient availability underscores its resilience and potential for spread in changing ecosystems [19].

Based on existing knowledge of the *Ambrosia* genus, we hypothesize that temperature influences how *A. confertiflora* allocates and distributes its resources. We created heat maps and growth models to show how the plant directs resources to different organs, supporting its rapid growth, adaptation, and establishment under changing climatic conditions. This knowledge can help develop effective management strategies to control and prevent the further spread of this highly invasive and allergenic species.

## 2. Materials and Methods

### 2.1. Seedling Preparation

Seeds of *A. confertiflora* were collected from plants along the Alexander riverbanks [6], sown and grown in a greenhouse under ambient conditions. The mother plants were allowed to cross with each other for at least two generations before use. Seeds germinated at 16/22 °C N-D 14 h daylight, in trays (50 × 30 × 6.5 cm) filled with a commercial growth medium, without any pre-germination treatment, were evenly spread on the moist soil surface to ensure optimal germination [8,9]. To ensure uniform growth, seedlings were selected at the four-leaf stage for uniformity and transplanted into containers according to the specific experimental setup.

### 2.2. Sprout Development and Position

An individual seedling at the four-leaf stage was transplanted to the center of a tray (50 × 30 × 6.5 cm) filled with a commercial growth medium. Ten trays (replicates) per treatment were placed in a phytotron under four temperature regimes (10–16 °C, 16–22 °C, 22–28 °C, 28–34 °C, N-D, 14 h daylight) at the Faculty of Agriculture, Rehovot, Israel. Sprouts, the new stems sprouting from the belowground rhizomes, were counted weekly for 160 days. Their positions were recorded using 10 × 8 cm grids to create qualitative heat maps showing the spatial distribution of sprouts relative to the initial seedling positioned in the tray center. The experiment was repeated twice.

### 2.3. Apical Dominance

To test apical dominance, individual *A. confertiflora* seedlings at the four-leaf stage were grown in trays (50 × 30 × 6.5 cm) as previously described, with 10 replicates per treatment. Trays were placed under four temperature regimes, as detailed above. At 74 DAP (days after planting), plants in five trays from each regime were clipped 5 cm above ground level. The remaining five trays were allowed to grow as before. The number of sprouts in both clipped and unclipped seedlings was recorded weekly until 140 DAP. The experiment was repeated twice.

### 2.4. Development of Shoot Height, Mass, and Underground Mass

Seedlings at the four-leaf stage were transplanted into 3.9 L plastic pots (18 cm in diameter) filled with commercial growth medium. Each pot contained one plant, with eight replicates per temperature regime. Sampling began at 35 DAP and continued at weekly intervals until 100 DAP. Five plants were cut at the soil surface, and shoot fresh and dry weight, as well as root and rhizome fresh and dry weights, were recorded following the careful removal of soil and rinsing the underground parts. Leaf area was measured with a flatbed scanner (Epson 12000XL, Seiko EPSON, Suwa-shi, Nagano, Japan), and analyzed with WinRHIZO Pro software vers 2017a, (Regent Instruments Ltd., Ontario, Canada).

To develop a thermal-growth model, all phenological data were linked to the corresponding temperature regime, and the biomass data were plotted versus Growing Degree Days (GDD). We used a function that averages the daily maximum and minimum temperatures, minus a T-base of 5.1 °C according to the following equation [20]:

(1) $$GDD=\sum_{=1}^{n}\left(\left(\frac{Tmax-Tmin}{2}\right)-Tbase\right)$$

T_max_ is the maximal daily temperature in °C, T_min_ the minimal daily temperature in °C, T_base_ is the base temperature in °C, below which no growth processes occur, and n represents the number of days.

We used a 3-parameter sigmoid model, which has been shown to effectively describe plant development [21,22]. This temperature-dependent model includes three stages: a Lag stage during which the plant develops at a moderate rate, a Log stage with rapid linear growth, and a saturation stage where growth levels off as it reaches its peak.

### 2.5. Statistical Analysis

All experiments were repeated twice using a completely randomized full factorial design, with varying numbers of replicates. Since there were no experiments examining treatment interaction, data from both runs were combined for analysis. Standard errors of the means are presented for each treatment. Differences between treatments were quantified by using an F distribution test at a significance level of *p* ≤ 0.05, using SigmaPlot 14.0 (Systat Software Inc., San Jose, CA, USA). The model used the equation below that describes the maximum asymptote (a); X_0_ as the inflection point, and b is the slope at the turning point.

(2) $$f\left(x\right)=\frac{a}{1+e^{\frac{x-xₒ}{b}}}$$

## 3. Results and Discussion

### 3.1. Sprout Development

**Figure 1:**
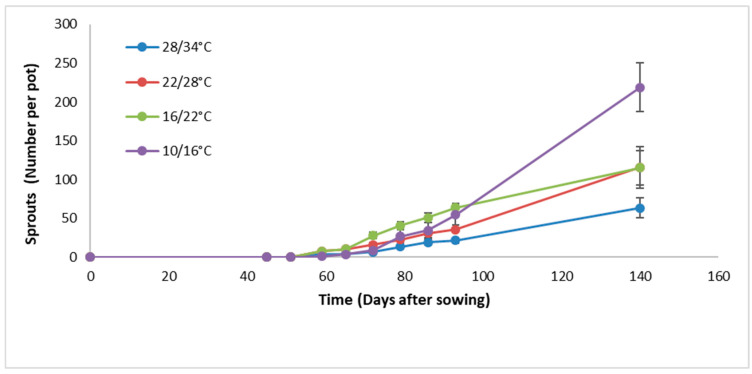
The number of *A*. *confertiflora* sprouts over time in trays under different temperature regimes in the phytotron greenhouse. Error bars represent standard error of the mean, *p* = 0.05.

At the lowest temperature regime (10/16 °C), sprouting began at 60 DAP, reaching 70 sprouts at 100 DAP, and an average of 250 sprouts per plant (100–350 per plant) at 140 DAP. In contrast, higher temperatures sharply reduced sprouting. Under the warmest temperature regime (28/34 °C) sprouting also began at 60 DAP, but only 16 sprouts were recorded by 100 DAP, and just 70 sprouts by 140 DAP (Figure 1). These results demonstrate that low temperatures significantly increase the sprouting rate of *A*. *confertiflora*, while high temperatures suppress it.

#### 3.1.1. Sprout Distribution

**Figure 2:**
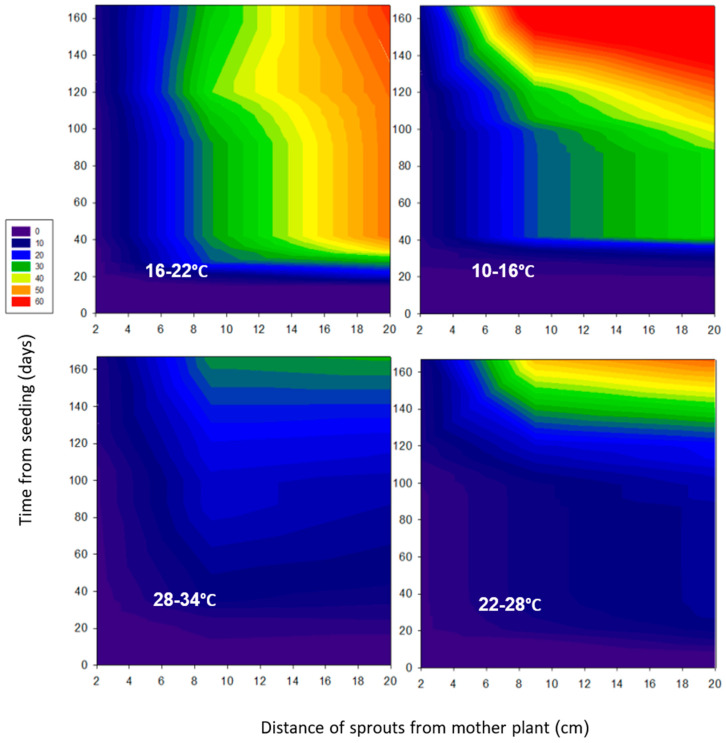
Heatmaps showing the spatial distribution of *A*. *confertiflora* sprouts over time under four temperature regimes in the phytotron. The mother plant is located at the intersection of the x- and y-axes. The color scale indicates the number of rhizomes per plant.

The spatial distribution of the sprouts is demonstrated in heat maps, which illustrate how sprouts expanded outwards from the mother plant over time. The mother plants are positioned at the x–y axes crossing point (Figure 2). Across all temperature regimes, sprouts tended to develop toward the edges of the tray. The highest number of sprouts occurred at 10/16 °C, whereas the fewest developed at 28/34 °C. Intermediate sprouting was observed at 22/28 °C and 16/22 °C, falling between the extremes of the coldest and warmest regimes.

#### 3.1.2. Apical Dominance

**Figure 3:**
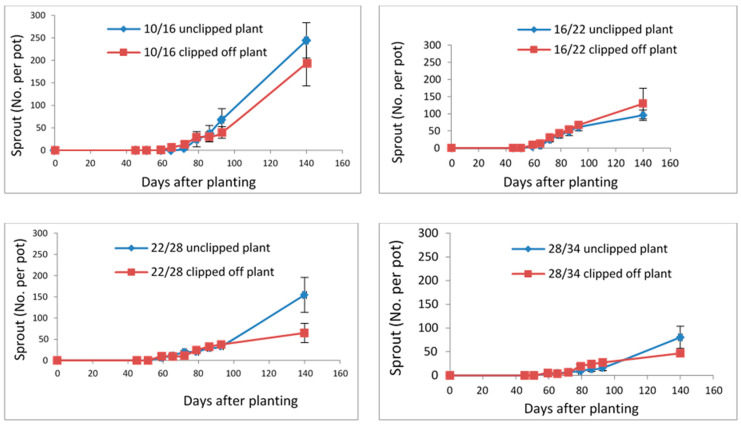
The effect of shoot apex clipping on the number of *A*. *confertiflora* sprouts under different temperature regimes. Clipping was performed at 74 days after planting (DAP). Error bars represent the standard error of the mean.

Apical dominance refers to the hormonal control exerted by the shoot apex, which suppresses the growth of lateral buds and slows sprouting. This control can also influence the orientation and growth of other plant organs, including leaves, branches, flowers, roots, rhizomes, stolons, and tubers [23,24]. As shown in Figure 3, removing the shoot apex (clipping) generally led to a reduction in the number of sprouts; however, this trend was not statistically significant. Cutting or mowing the apex of *A*. *confertiflora* did not significantly affect sprouting across any of the temperature regimes. Sprouting continued to follow the same temperature-dependent pattern—highest at the lowest temperature (10/16 °C) and lowest at the highest temperature (28/34 °C)—regardless of whether the apex was removed.

### 3.2. Thermal Modeling

**Figure 4:**
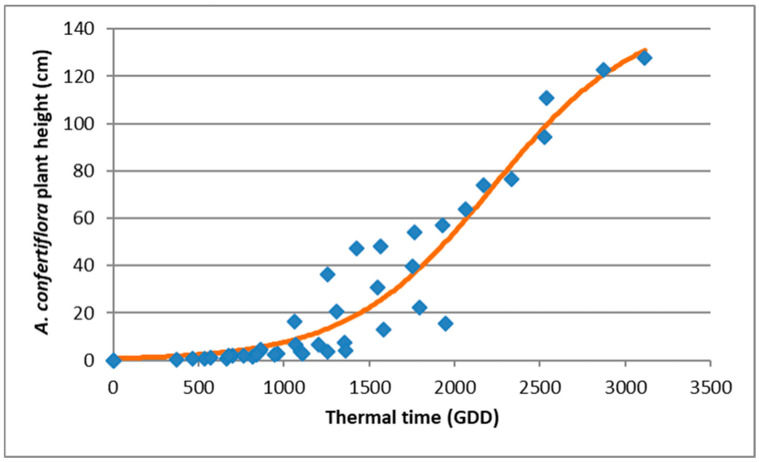
*Ambrosia confertiflora* height as a function of accumulated Growing Degree Days (GDD) in the phytotron. Data points represent observed plant heights, and the regression line was plotted using Equation (3), which models shoot elongation based on GDD.

To evaluate the effect of temperature on *A. confertiflora* growth and development, we used a thermal model based on the methods described in [21,22], We measured shoot height, aboveground biomass, and underground biomass and analyzed these variables in relation to accumulated Growing Degree Days (GDD) (Figure 4). The base temperature (T-base) for plant development was determined to be 5.1 °C [20]. GDD was calculated using the following Equation (3), which describes *A. confertiflora* elongation.

Y = 146.7/1 + exp(−(x − 222.75)/422.53)) (*p* < 0.0001) (3)

**Figure 5:**
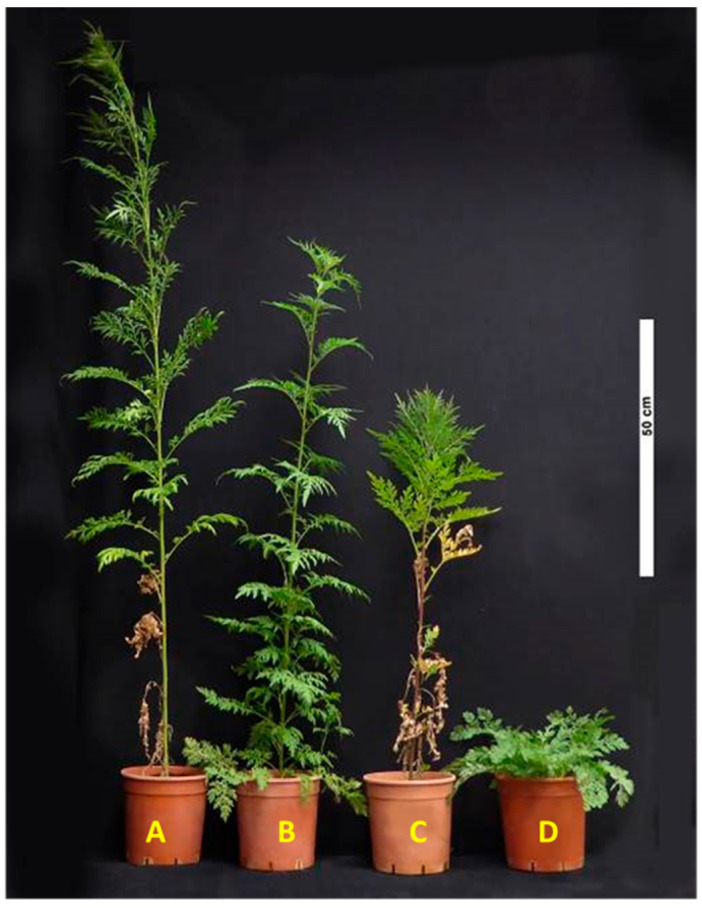
Effect of temperature regimes on *A*. *confertiflora* elongation at 83 DAP. (**A**). Plant grown continuously at 28/34 °C. (**B**). Plant grown at 16/10 °C until 49 DAP, then transferred to 28/34 °C for 34 days. (**C**). Plant grown at 28/34 °C until 49 DAP, then transferred to 10/16 °C for 34 days. (**D**). Plant grown continuously at 10/16 °C.

As previously noted, plant height in *A. confertiflora* is temperature-dependent. This relationship was further confirmed in a dedicated experiment. Plants grown in pots under the 22/28 °C and 28/34 °C regimes reached an average height of 1.80 m, compared to their natural height of 3.0–4.0 m. In contrast, plants grown at the lower temperature regimes (10/16 °C and 16/22 °C) reached only about 5 cm in height (Figure 5).

**Figure 6:**
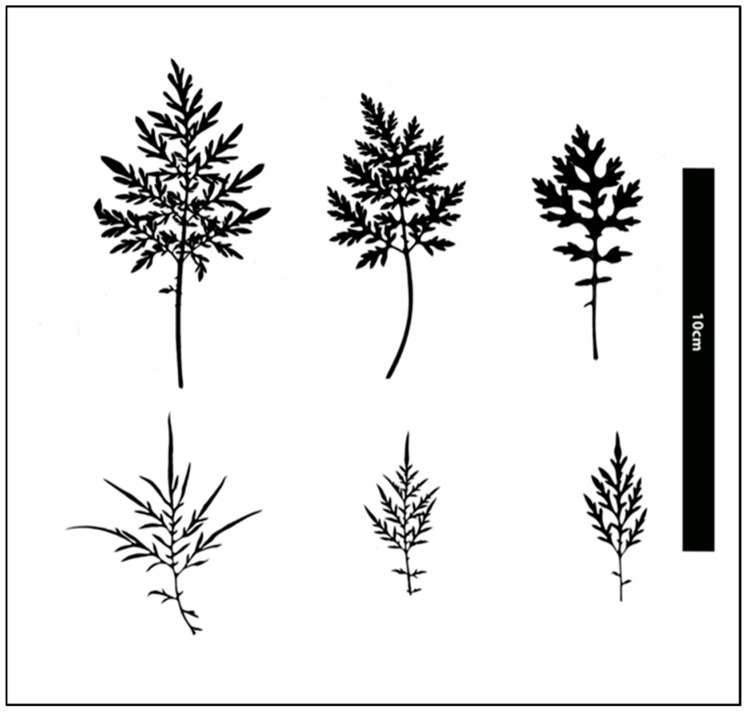
Scanned images of mature *A*. *confertiflora* leaves at 85 DAP, taken from the second leaf below the apex using a flatbed scanner (Epson 12000XL, Seiko EPSON, Japan). The top row shows leaves from three plants grown at 10/16 °C, while the bottom row shows leaves from three plants grown at 28/34 °C. Scale bar = 10 cm.

Although the number of leaves remained similar across temperature treatments, lower temperatures strongly suppressed internode elongation. As shown in Figure 5, plant elongation corresponded with accumulated GDD in the different tested temperature regimes. By 100 DAP, plants grown at 10/16 °C accumulated 1400 GDD, those at 16/22 °C reached 1950 GDD, and plants at 28/34 °C accumulated 3100 GDD. Most plants grown under the coldest regime (10/16 °C regime, 1400 GDD) developed a rosette, with minimal elongation not exceeding 20 cm. In contrast, plants that accumulated more than 1600 GDD showed significant shoot elongation, indicating that sufficient heat accumulation is required for vertical growth (Figure 5 and Figure 6).

In an additional pot experiment, *A*. *confertiflora* plants were transferred at 49 DAP between the coldest (10/16 °C) and warmest (28/34 °C) temperature regimes. Plants moved from the warm regime to the cold regime developed new leaves in a rosette form. In contrast, those transferred from the cold to the warm regime commenced normal stem elongation (Figure 5).

**Figure 7:**
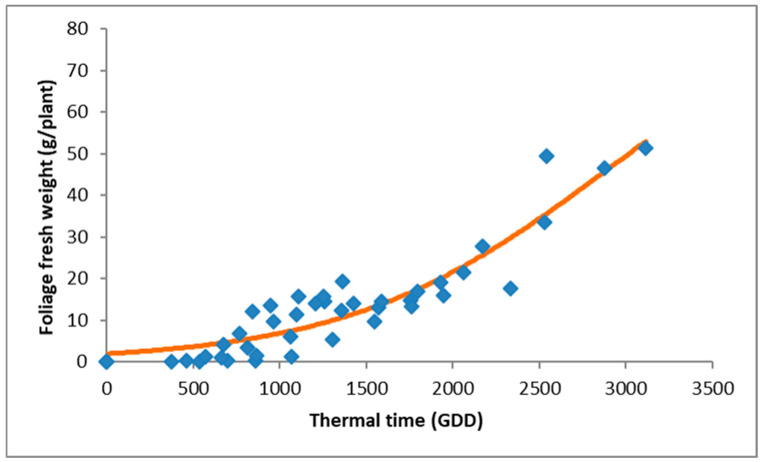
Accumulation of *A*. *confertiflora* shoot fresh weight in response to thermal time (GDD) in the phytotron. Data points represent observed values, and the curve was fitted using Equation (4), which models biomass accumulation based on GDD.

Although plant height was much lower in the cold regime compared to the warm regime, plants grown at 10/16 °C developed a leaf area 2.75 times larger than those grown at 28/34 °C, 67 cm^2^ versus 25 cm^2^ per leaf area, respectively. (Figure 6). Additionally, in the coldest regime, plants produced longer petioles and broader leaves compared to those in the warmest regime (Figure 7). The reduction in leaf size at higher temperatures is a typical plant adaptation to dry conditions, helping to minimize water loss through evapotranspiration [25].

### 3.3. Shoot Biomass

*Ambrosia confertiflora* plants grown under all four temperature regimes in the phytotron began to accumulate shoot mass only after accumulating at least 1000 GDD. As GDD increased, shoot mass continued to rise proportionally (Figure 7). This phenomenon differs from the shoot elongation response described in Figure 4. The accumulation of shoot mass is described in Equation (4):

Y = 91.34/(1 + exp(−(x − 2874.97)/746.66)) (*p* < 0.0001): (4)

### 3.4. Underground Biomass Accumulation

Underground biomass in *A*. *confertiflora* developed differently under the four temperature regimes. Unlike shoot biomass, no single regression line or equation could describe the relationship between underground mass and GDD. Therefore, a separate equation (Equation (5)) was fitted for each temperature regime.

**Figure 8:**
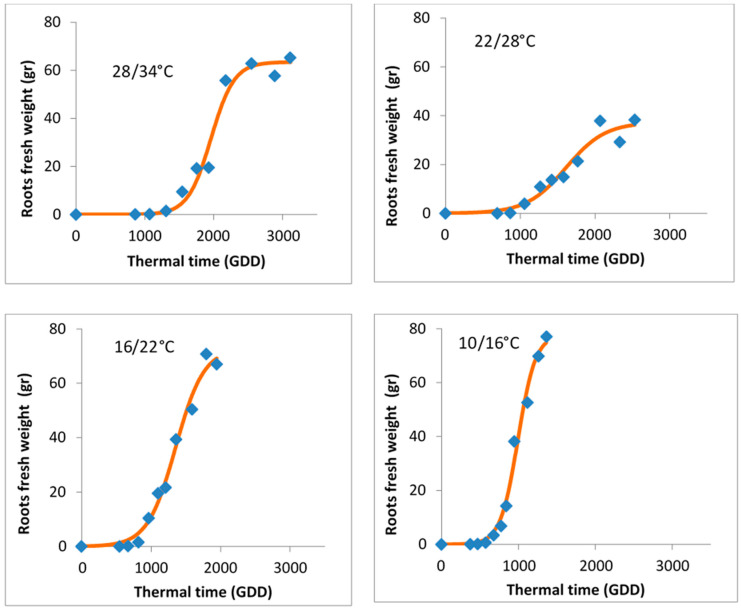
Accumulation of *A*. *confertiflora* underground biomass in response to thermal time (GDD) across four temperature regimes: 28/34 °C, 22/28 °C, 16/22 °C, and 10/16 °C. Data points represent observed values, and individual regression lines were fitted by using Equation (5) for each temperature regime.

In the coldest regime (10/16 °C), underground mass accumulated rapidly. As temperatures increased, the rate and total accumulation of underground mass decreased (Figure 8).

Equation (5):

(5) $$\begin{array}{c} Y=a/(1+\exp(-(x-x0)/b))\\ \mathrm{If}\;34>x>28\\ Y=63.44/(1+\exp(-(x-1961.3)/167.8))\;(p<0.0001)\\ \mathrm{If}\;28>x>22\\ Y=37.35/(1+\exp(-(x-1609.7)/264.13))\;(p<0.0001)\\ \mathrm{If}\;22>x>16\\ Y=72.96/(1+\exp(-(x-1351.37)/203.95))\;(p<0.0001)\\ \mathrm{If}\;16>x>10\\ Y=77.87/(1+\exp(-(x-992.2)/114.84))\;(p<0.0001) \end{array}$$

**Figure 9:**
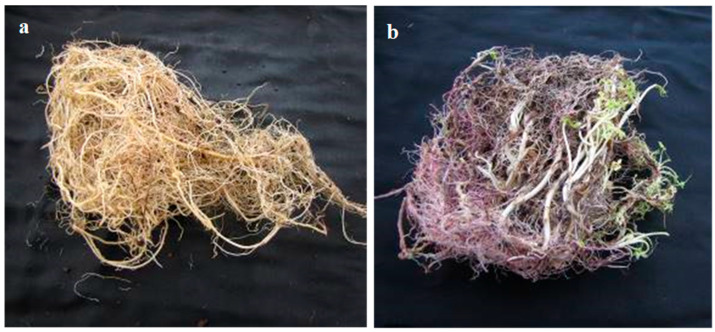
Underground structures of *A*. *confertiflora* (roots and rhizomes) at 98 DAP, grown under two temperature regimes: (**a**) 28/34 °C; (**b**) 16/10 °C.

These results are unexpected, as the GDD model was anticipated to normalize temperature effects on the development of underground organs, as demonstrated for plant height and foliage biomass accumulation. We suggest that the variation in underground biomass accumulation reflects different resource allocation strategies based on environmental needs. At low temperatures, plants appear to prioritize rhizome and sprout formation to secure sufficient carbon assimilation through the rosette. In contrast, at higher temperatures, resources are directed primarily toward root development to ensure water supply for the developing shoot system. This interpretation is supported by the observed increase in sprouting and the formation of thinner roots under lower temperatures (Figure 9).

### 3.5. Biomass Allocation

Previous studies [8,9] reported that *A. confertiflora* seeds exhibit high germination rates (up to 80%) without requiring dormancy-breaking treatments. Germination occurs on the soil surface in the presence of light. Young seedlings remain susceptible to control measures for approximately the first 60 days, before significant underground organ development begins.

Resource allocation in *A. confertiflora* depends on both temperature and developmental stage. Lower temperatures favor investment in underground biomass, mainly rhizomes, for establishment. As temperatures rise, allocation shifts towards stem elongation, flowering, and root formation. This ontogenetic pattern progresses from initial investment in foliage, followed by underground structures, and eventually leads to a balanced distribution between above- and belowground biomass at maturity.

The temperature regimes tested in this study correspond to seasonal averages in Israel. *A. confertiflora*’s response to temperature aligns with its natural phenological cycle: germination and establishment occur in late winter and spring; stem elongation follows in spring and summer; flowering begins in late summer and fall; and seed production peaks in late fall and early winter. During winter, germinating plants form a rosette aboveground without stem elongation. In contrast, hot, dry summer conditions suppress germination, which resumes as temperatures fall and winter rains return.

**Figure 10:**
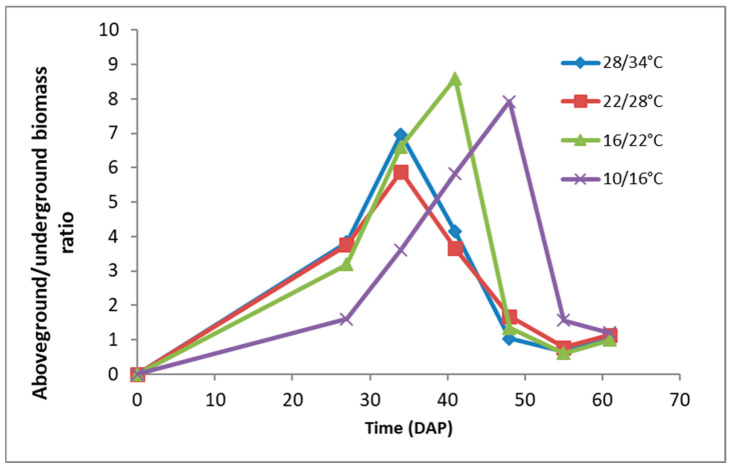
Ratio aboveground (foliage) to underground (roots and rhizomes) fresh weight in *A*. *confertiflora* plants at 60 DAP, grown under different temperature regimes in the phytotron.

**Figure 11:**
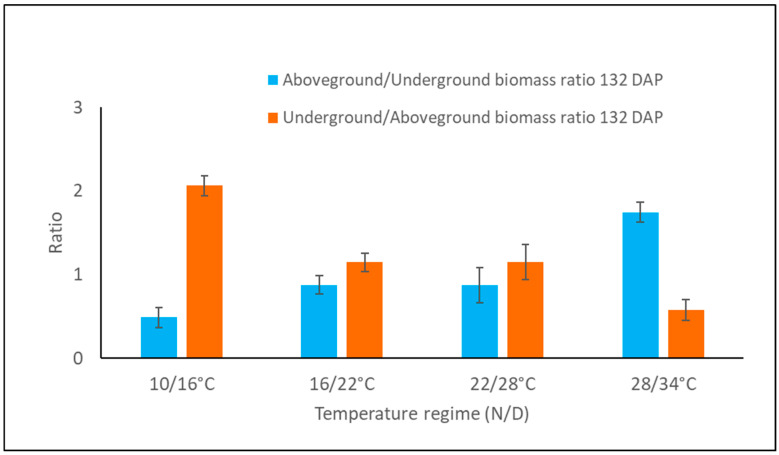
Ratio of aboveground to underground biomass in *A. confertiflora* grown for 132 DAP under different temperature regimes. Bars denote standard error of the mean.

Notably, *A. confertiflora* exhibits up to a ninefold increase in the foliage-to-root ratio during development, with this growth pattern more pronounced under lower temperature regimes (16/22 °C and 10/16 °C). This suggests an early emphasis on photosynthetic capacity. By 55–60 DAP, foliage-to-root ratio approaches 1 (Figure 10), followed by a rapid increase in root and rhizome biomass production, especially at 10/16 °C. By 132 DAP, belowground biomass exceeded foliage mass by more than twofold (Figure 11). This temperature-driven allocation strategy likely enhances the plant’s competitive ability for resources, aiding in its spatial dominance and invasive success.

Under high temperatures, *A. confertiflora* development proceeds through several distinct growth phases (Figure 10):

1. **Establishment** (up to 10 DAP)**:** Balanced resource allocation between foliage and underground mass.
2. **Foliage Development** (25 to 50 DAP)**:** Prioritization of aboveground growth to maximize photosynthesis, regardless of plant height and stem elongation.
3. **Underground Investment** (35 to 55 DAP)**:** Allocation shifts toward the development of roots and rhizomes, supported by earlier foliage growth.
4. **Balanced Allocation** (from 60 DAP)**:** Resources are distributed equally between above- and belowground systems.

This sequential pattern reflects a temperature- and stage-dependent strategy that supports both early establishment and long-term persistence. The flexibility in resource allocation under varying thermal conditions highlights the species’ phenotypic plasticity, which likely contributes to its invasive potential and ecological success. These findings provide a physiological framework for understanding its spread and inform strategies for its management.

## 4. Conclusions

This study provides new insights into the invasion dynamics, ecological impact, and morphological development of *A. confertiflora,* offering an explanation for its rapid dissemination and detrimental effects on agri-ecosystems. The findings highlight the species’ phenotypic plasticity and its response to temperature variation. Temperature plays a central role in shaping the development of both aboveground and underground plant organs. At lower temperatures (during winter), *A. confertiflora* allocates more resources to underground growth, particularly rhizome formation. At higher temperatures, resource allocation shifts toward shoot and leaf development. This temperature-driven strategy enables the plant to adapt to a wide range of conditions. This unique behavior could also serve as an indication for weed management timing, e.g., by aiming the application of control measures at the rosette growing stage, hence reducing rhizome proliferation during the cold temperatures.

Our observation indicates that this typical behavior cannot be detected in a closely related weed—*A. teneifolia*, a perennial, naturalized weed in Israel [10], which does not form a rosette in the winter, but rather continues with its normal growth even in the Israeli winter. Biomass allocation in *A. confertiflora* also varies with developmental stage. During the growing season, energy is directed primarily to aboveground structures, and as the plant matures, there is a shift towards underground structures until a balanced distribution is achieved.

*Ambrosia confertiflora* demonstrates high phenotypic adaptability in response to temperature changes, influencing traits such as plant height and leaf morphology. These adaptive traits are key to its establishment and expansion in diverse environments. Understanding how temperature influences the development and spread of *A*. *confertiflora* is critical for assessing its ecological impact and designing effective management strategies. The knowledge gained from this research can lead to targeted weed management strategies to curb the spread of this highly invasive and allergenic species.

## Data Availability

The data presented in this study are openly available in [Baruch Rubin] at rubin@mail.huji.ac.il.
